# Bifunctional Malic/Malolactic Enzyme Provides a Novel Mechanism for NADPH-Balancing in Bacillus subtilis

**DOI:** 10.1128/mBio.03438-20

**Published:** 2021-04-06

**Authors:** Manuel Hörl, Tobias Fuhrer, Nicola Zamboni

**Affiliations:** a Institute of Molecular Systems Biology, ETH Zürich, Zürich, Switzerland; University of Geneva

**Keywords:** bifunctional enzyme, redox metabolism, NADPH balance, ^13^C metabolic flux analysis, malic enzyme, malolactic enzyme, metabolism

## Abstract

The redox cofactor NADPH is required as a reducing equivalent in about 100 anabolic reactions throughout metabolism. To ensure fitness under all conditions, the demand is fulfilled by a few dehydrogenases in central carbon metabolism that reduce NADP^+^ with electrons derived from the catabolism of nutrients. In the case of Bacillus subtilis growing on glucose, quantitative flux analyses indicate that NADPH production largely exceeds biosynthetic needs, suggesting a hitherto unknown mechanism for NADPH balancing. We investigated the role of the four malic enzymes present in B. subtilis that could bring about a metabolic cycle for transhydrogenation of NADPH into NADH. Using quantitative ^13^C metabolic flux analysis, we found that isoform YtsJ alone contributes to NADPH balancing *in vivo* and demonstrated relevant NADPH-oxidizing activity by YtsJ *in vitro*. To our surprise, we discovered that depending on NADPH, YtsJ switches activity from a pyruvate-producing malic enzyme to a lactate-generating malolactic enzyme. This switch in activity allows YtsJ to adaptively compensate for cellular NADPH over- and underproduction upon demand. Finally, NADPH-dependent bifunctional activity was also detected in the YtsJ homolog in Escherichia coli MaeB. Overall, our study extends the known redox cofactor balancing mechanisms by providing first-time evidence that the type of catalyzed reaction by an enzyme depends on metabolite abundance.

## Abstract (precis)

A new mechanism for NADPH balancing was discovered in Bacillus subtilis. It pivots on the bifunctional enzyme YtsJ, which is known to catalyze NADP-dependent malate decarboxylation. We found that in the presence of excessive NADPH, the same enzyme switches to malolactic activity and creates a transhydrogenation cycle that ultimately converts NADPH to NADH. This provides a regulated mechanism to immediately adjust NADPH/NADP^+^ in response to instantaneous needs.

## INTRODUCTION

Bacteria grow in various environments by metabolizing a plethora of small carbon compounds, such as sugars, alcohols, and organic acids. These substrates are catabolized by the approximately 60 reactions of central carbon metabolism and converted into a small set of precursors sufficient to synthesize all cellular components. In parallel to abridging catabolism and anabolism, central carbon metabolism is also the prime source for energy and reducing power to enable all cellular functions. This is accomplished by either investing electrons obtained from oxidative catabolism for the phosphorylation of ADP to ATP (1, 2) (directly or via NADH and the electron transport chain) or allocating them on the redox cofactor NADPH, which serves as the main reducing equivalent to drive anabolism or fight oxidative stress (3, 4). At a cellular level, organic cofactors such as ATP and NADPH are continuously used by a multitude of kinases and dehydrogenases. A drop in cofactor levels causes ubiquitous complications; therefore, swift recycling by central carbon metabolism is critical to ensure fitness or mitigate damage in the face of oxidative stress (5–7).

In the case of NADPH, the primary producers are glucose-6-phosphate and 6-phosphogluconate dehydrogenase in the oxidative pentose phosphate pathway (PPP) and isocitrate dehydrogenase in the tricarboxylic acid (TCA) cycle. Many organisms also contain NADPH-dependent malic enzymes (8–11). The production of NADPH by these enzymes is stoichiometrically coupled to their carbon flux; therefore, it is necessary to coordinate across carbon, redox, and energy networks. Microorganisms have evolved different strategies to optimize this interplay. Yeasts are indeed able to adjust their intracellular rates such that NADPH production and demand are balanced (12, 13). In contrast, bacteria tend to produce more NADPH than required for anabolism (14) but have developed means to exchange electrons with NAD^+^/NADH and thereby compensate for a deficit or excess in NADPH. Two such biochemical mechanisms have been reported. First, organisms may possess nicotinamide nucleotide transhydrogenases, which catalyze the reversible transfer of electrons between NAD(H) and NADP(H) (15). Their physiological role in NADPH metabolism has been studied in detail in Escherichia coli, where the membrane-bound, energy-dependent transhydrogenase PntAB reduces NADP^+^ to compensate for NADPH underproduction, while the soluble transhydrogenase UdhA oxidizes NADPH under excess production (5). Second, biochemical redox cycles consisting of two isoenzymes with different cofactor specificities that operate in a cyclic manner can realize a net transhydrogenation from NADPH to NADH (or vice versa) without affecting net carbon fluxes. Examples of such redox cycles include the simultaneous operation of isoforms of isocitrate dehydrogenase in animal mitochondria (16) and alcohol and glyceraldehyde-3-phosphate dehydrogenases in Kluyveromyces lactis (17).

Although the aforementioned mechanisms allowed us to quantitatively understand NADPH metabolism in many microbes, there are still species for which it is not possible to reconcile NADPH balancing. A prominent example is the Gram-positive bacterium Bacillus subtilis, which consistently exhibits extensive NADPH overproduction during growth on glucose (14, 18). Neither a transhydrogenase nor a transhydrogenation cycle has been identified so far that closes the gap. Two such potential cycles exist in the central metabolism of B. subtilis. The first cycle is formed by the NAD^+^- and NADP^+^-specific glyceraldehyde-3-phosphate dehydrogenases GapA and GapB, operating in opposite directions (19). However, it is well established that GapB is under strong catabolite repression during growth on glucose (20, 21). The second putative cycle might include the NADPH-dependent malic enzyme YtsJ and one or more of the three B. subtilis malic enzymes that prefer NADH (MaeA, MalS, and MleA) (3, 41). Together, these could create a transhydrogenation cycle (19) that compensates for the apparent NADPH overproduction by transferring excess electrons to NADH and, eventually, the electron transfer chain.

Here, we set out to investigate the role of the B. subtilis malic enzyme isoforms in NADPH balancing during growth on glucose. We quantified intracellular fluxes and NADPH balances in several B. subtilis malic enzyme deletion strains using both stationary and nonstationary ^13^C-labeling experiments. Flux analysis and *in vitro* data indicated that YtsJ indeed oxidizes NADPH but not in a transhydrogenation cycle with other malic enzymes. We further investigated the specific mechanism by combining *in vitro* enzymatic assays with untargeted metabolite profiling data. This led to the discovery of a second, redox-neutral malolactic reaction of YtsJ, which was only active in the presence of excess NADPH and would allow for its balancing.

## RESULTS

### The cellular NADPH balance changes upon deletion of malic enzyme YtsJ.

To investigate the role of malic enzymes for NADPH balancing in B. subtilis, we used ^13^C metabolic flux analysis to quantify NADPH production and consumption in wild-type and malic enzyme Δ*maeA*, Δ*malS*, Δ*mleA*, and Δ*ytsJ* single-deletion mutants. Cells were cultured either with 100% [1-^13^C]glucose or a mixture of 50% naturally labeled and 50% [U-^13^C]glucose. At mid-exponential phase, cells were harvested and hydrolyzed. ^13^C labeling patterns of protein-bound amino acids by gas chromatography-mass spectrometry (GC-MS) analysis were used to calculate ratios of converging metabolic fluxes (22). These ratios, together with physiological rates, were then used to constrain a stoichiometric model of B. subtilis central metabolism (23) to calculate intracellular flux rates.

**FIG 1:**
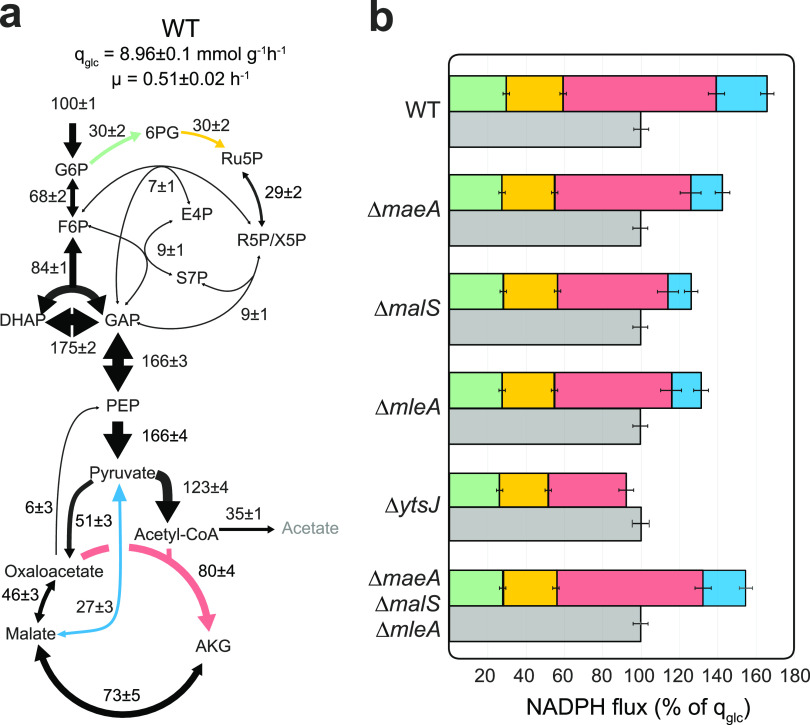
Cellular NADPH balance. (a) Metabolic flux distribution in B. subtilis wild type, estimated by ^13^C flux ratio analysis and metabolite balancing. Flux values are normalized to the glucose uptake rate (*q*_glc_; mmol gCDW^−1^ h^−1^), and arrow sizes scale with flux magnitudes. (b) NADPH balances of wild-type and malic enzyme deletion strains. NADPH production is calculated by summing the flux rates of NADPH-dependent glucose-6-phosphate dehydrogenase (green), 6-phosphogluconate dehydrogenase (yellow), isocitrate dehydrogenase (red), and NADP^+^ specific malic enzyme (blue) and compared to normalized growth-dependent NADPH consumption (gray).

The results for wild-type B. subtilis favorably matched previously reported results (18, 24), with glucose being catabolized by glycolysis and the pentose phosphate pathway at a ratio of ∼70%:30%, respectively (Fig. 1a). Glucose was further catabolized through the TCA cycle, being either oxidized to CO_2_ or incorporated into biomass, with only a small fraction being secreted as acetate. Deletion of each malic enzyme isoform provoked a slight reduction in growth rate and an increase in acetate yield by up to 15% (see Table S1 in the supplemental material). Estimated fluxes exhibited and lower flux through the TCA cycle and the malic enzyme reaction, while fluxes in glycolysis and the pentose phosphate pathway were almost identical (Fig. S1a to d). To estimate the NADPH balancing capacities of each strain, we summed the contribution of known NADPH-producing reactions, including the hypothetical NADPH formation by the malic enzyme YtsJ, since it has a strong preference for NADP^+^ and is the most active isoenzyme during growth on glucose (8). Total NADPH reduction was compared to that of growth-coupled NADPH oxidation to obtain a balance (Fig. 1b). Consistent with previous studies (14), wild-type B. subtilis exhibited an apparent NADPH overproduction of 67% compared to cellular requirements. NADPH overproduction was roughly halved in Δ*maeA*, Δ*malS,* and Δ*mleA* mutants and virtually abolished in the Δ*ytsJ* mutant. This reduction was the result of lower fluxes through the malic enzyme and the TCA cycle by isocitrate dehydrogenase (red and blue bars in Fig. 1b). Deletion of YtsJ caused a rearrangement of fluxes in which NADPH reduction and oxidation are balanced. Therefore, we speculated that the primary function of YtsJ during growth on glucose was not the generation of reduced NADPH or pyruvate but contributing to oxidation of excess NADPH produced by other enzymatic reactions.

10.1128/mBio.03438-20.1 FIG S1 Metabolic flux distributions in B. subtilis malic enzyme deletion mutants, estimated by ^13^C flux ratio analysis and metabolite balancing. Flux values are normalized to the glucose uptake rate (*q*_glc_; mmol g_CDW_^−1^ h^−1^) of each strain. Arrow dimensions scale with flux magnitude. Download FIG S1, PDF file, 0.3 MB. Copyright © 2021 Hörl et al. 2021 Hörl et al. https://creativecommons.org/licenses/by/4.0/ This content is distributed under the terms of the Creative Commons Attribution 4.0 International license.

10.1128/mBio.03438-20.4 TABLE S1 Physiological parameters of the six B. subtilis strains used in this study. The mean values and standard deviations were determined from at least duplicate experiments. Download Table S1, PDF file, 0.1 MB. Copyright © 2021 Hörl et al. 2021 Hörl et al. https://creativecommons.org/licenses/by/4.0/ This content is distributed under the terms of the Creative Commons Attribution 4.0 International license.

### YtsJ is sufficient to consume excess NADPH.

**FIG 2:**
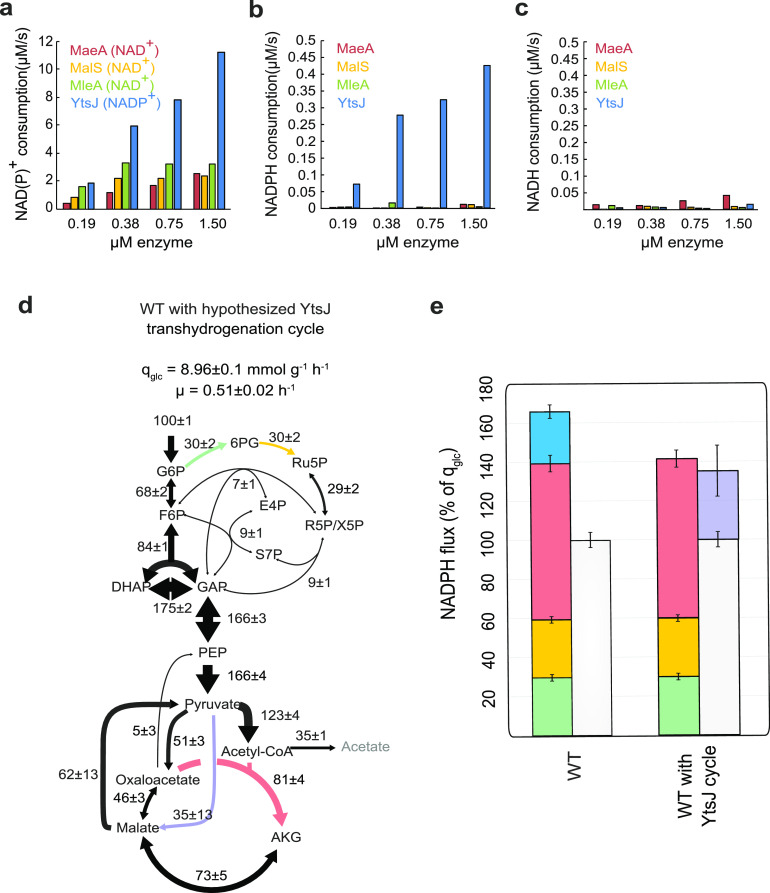
NADPH oxidation by malic enzyme. (a to c) Initial reaction rates of purified MaeA, MalS, MleA, and YtsJ at different enzyme concentrations. (a) Oxidative malate decarboxylation rate in the presence of each enzymes’ preferred cofactor. (b and c) Reductive pyruvate carboxylation rate with NADPH (b) and NADH as cofactor (c). (d) Metabolic flux distribution in B. subtilis wild type with hypothetical NADPH-oxidizing cycle, estimated from stationary and nonstationary ^13^C flux data. (e) NADPH balance in B. subtilis wild type with and without the hypothetical, NADPH-oxidizing cycle. Glucose-6-phosphate dehydrogenase (green), 6-phosphogluconate dehydrogenase (yellow), isocitrate dehydrogenase (red), and NADP^+^ specific malic enzyme (blue) compared to the normalized growth-dependent NADPH consumption (white) and YtsJ cycle (purple).

To demonstrate that YtsJ is indeed capable of catalyzing NADPH oxidation, we performed *in vitro* biochemical assays with all four malic enzymes purified with a His-Tag upon overexpression in E. coli (8). We verified that activity was preserved after purification by measuring malate decarboxylation activity with the preferred cofactor (8), using a spectrophotometric assay (14) (Fig. 2a). We then assayed the reverse reaction of reductive pyruvate carboxylation for all combinations of the four isoenzymes and either NADH or NADPH. Out of all pairs, we detected substantial reductive activity only with YtsJ and NADPH (Fig. 2b and c). To verify physiological relevance, we determined key kinetic parameters for YtsJ (Table S2). The *K_m_* values for both pyruvate (*K_m_*, 5.3 ± 1.3 mM) and NADPH (*K_m_*, 0.8 ± 0.5 mM) were found in the range of reported intracellular concentrations (pyruvate, 8.9 ± 5.75 mM; NADPH, 0.36 ± 0.25 mM) (14, 25). This indicated that the reverse reaction of pyruvate decarboxylation coupled to NADPH oxidation can also occur *in vivo*.

10.1128/mBio.03438-20.5 TABLE S2 Enzymatic parameters of the four B. subtilis malic enzyme. *K_m_* values are provided in mM, and *k*_cat_ values are in s^−1^. The mean values and standard deviations were determined with at least 2 replicates. Download Table S2, PDF file, 0.2 MB. Copyright © 2021 Hörl et al. 2021 Hörl et al. https://creativecommons.org/licenses/by/4.0/ This content is distributed under the terms of the Creative Commons Attribution 4.0 International license.

If the mechanism of YtsJ-mediated NADPH balancing was a transhydrogenation cycle formed by NADPH-consuming YtsJ and either one of the three NAD-dependent malic enzymes, the concomitant deletion mutant of all three NAD-dependent malic enzymes should cause a phenotype similar to that of the YtsJ knockout strain. However, intracellular fluxes in the Δ*maeA* Δ*malS* Δ*mleA* triple deletion mutant were similar to those of the wild-type (Fig. 1b and Fig. S1e), indicating that the presence of any of the NAD-dependent isoenzymes is not necessary for a transhydrogenation cycle composed solely of malic enzymes. We concluded that YtsJ alone or in combination with another, undiscovered mechanism endows the cell with a mechanism to oxidize excess NADPH.

One alternative mechanism is that the YtsJ-catalyzed reaction was highly reversible and that the enzyme creates transhydrogenation itself by oxidizing NADPH through pyruvate carboxylation but preferably reducing NAD^+^ instead of NADP^+^ through malate decarboxylation. Biochemically, this is possible because YtsJ can also use NAD^+^ as a cofactor for malate decarboxylation (EC 1.1.1.40) (8). Thermodynamically, transhydrogenation by YtsJ alone would only work if both reactions have negative (or close to equilibrium) Gibbs free energy (Δ_r_*G*). Based on the *in vivo* concentrations (Table S2 and Materials and Methods), we calculated the Δ_r_*G* (26) of NAD^+^-dependent malate decarboxylation to be −11 ± 6 kJ/mol. The Δ_r_*G* of NADPH-dependent pyruvate carboxylation was 6 ± 8 kJ/mol and, thus, close to equilibrium. To conclude, transhydrogenation by YtsJ is kinetically and thermodynamically plausible.

To quantify the potential impact of a reversible malic enzyme as a standalone transhydrogenation cycle on cofactor balancing, it is necessary to measure the flux in both directions. The measurement of the forward flux from malate to pyruvate requires nonstationary ^13^C experiments and was obtained from a previous study with identical conditions (27). We calculated the reverse flux by subtracting the net flux calculated in this study from stationary ^13^C experiments from published forward flux. Assuming that pyruvate carboxylation consumed NADPH and malate decarboxylation forms NADH, the NADPH balance for wild-type B. subtilis would be closed (Fig. 2d and e). This quantitative analysis suggests that YtsJ supports a transhydrogenation cycle that does not depend on other malic enzymes.

### Increasing NADPH levels transform YtsJ into a malolactic enzyme.

**FIG 3:**
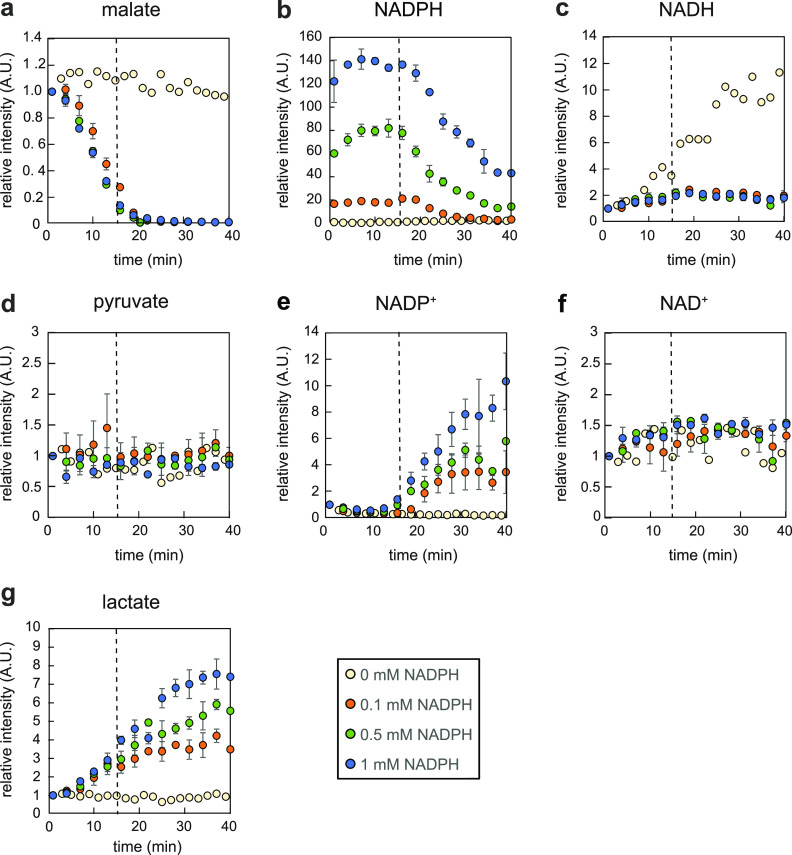
*In vitro* transhydrogenation assays with malic enzyme YtsJ. Time courses of relative intensities of metabolites at different NADPH concentrations present in the assay, recorded by direct flow injection analysis. Error bars represent deviations calculated from two independent assays.

To experimentally validate the proposed transhydrogenation, we assayed *in vitro* if increasing concentrations of NADPH would trigger increased NADH generation by YtsJ. Purified YtsJ was incubated at room temperature with a constant mix of NAD^+^, malate, and pyruvate at physiological concentrations and various NADPH amounts. Since NADH and NADPH cannot be distinguished by optical absorption, reactant levels were monitored by flow injection electrospray–time-of-flight mass spectrometry (TOF MS) (28). By accurate mass measurements, all reactants could be distinguished and tracked over time (Fig. 3). In the absence of NADPH, YtsJ catalyzed malate decarboxylation to pyruvate, indicated by a subtle decline in the malate signal and synchronous NADH formation (Fig. 3a and c). Notably, we expected pyruvate to remain constant regardless of the enzymatic activity because of its high concentration (∼9 mM) and detector saturation. Importantly, the temporal profiles changed substantially in the presence of NADPH. Malate consumption accelerated drastically until full depletion after 20 min (Fig. 3a). To our surprise, this rapid and immediate decrease in malate was not coupled to the formation of NADH or NADPH (Fig. 3b and c), leaving the question of how malate conversion could proceed if not through NAD(P)^+^-dependent decarboxylation. Just before malate was depleted, NADPH levels started to decline (Fig. 3b) and NADP^+^ to rise (Fig. 3e). Strikingly, NAD^+^ and NADH levels were constant (Fig. 3c and f). To summarize, YtsJ catalyzed NADPH oxidation and malate consumption independent of NAD^+^ reduction. This confuted the hypothesized transhydrogenation cycle and suggested the presence of a different, hitherto unknown mechanism.

To reveal the nature and stoichiometry of malate degradation, we searched, in the untargeted mass spectrometry data, for ions that correlated positively or negatively with the malate profile. We performed unsupervised k-means clustering on the 107 ion traces recorded by mass spectrometry for the assay with 1 mM NADPH (Fig. S2). Three ion clusters were identified. With very few exceptions, the majority of ions were constant and grouped in cluster 2. Cluster 3 contained ions that were consumed over time. These were all malate derivatives (i.e., isotopes and water loss). Cluster 1 contained the only 2 traces that increased over time, which could be associated with NADP^+^ and lactate. Lactate identity was confirmed by targeted liquid chromatography-tandem MS (LC-MS/MS) analysis of the reaction mixture (29) (Fig. S3). Expanding the analysis to all assays, we observed that lactate formation occurred from the very beginning but exclusively in the presence of NADPH in the reaction mix (Fig. 3g).

10.1128/mBio.03438-20.2 FIG S2 k-means clustering of the log-transformed, annotated ion responses of both assays with 1 mM NADPH, normalized to the initial time point. Three clusters were specified, and ions were assigned to the clusters based on squared Euclidean distance. The black line represents the centroid of each cluster. Cluster 1 of strongly increasing ions contained lactate and NADP^+^. Cluster 3 of strongly decreasing ions contained malate as a deprotonated (*m/z* 133) ion and an ion with neutral loss of water (*m/z* 115). Download FIG S2, PDF file, 0.2 MB. Copyright © 2021 Hörl et al. 2021 Hörl et al. https://creativecommons.org/licenses/by/4.0/ This content is distributed under the terms of the Creative Commons Attribution 4.0 International license.

10.1128/mBio.03438-20.3 FIG S3 Confirmation of ion *m/z* 89 in enzyme assay mixture as lactate by multiple-reaction monitoring analysis (89→43 transition) using targeted LC-MS/MS. Download FIG S3, PDF file, 0.2 MB. Copyright © 2021 Hörl et al. 2021 Hörl et al. https://creativecommons.org/licenses/by/4.0/ This content is distributed under the terms of the Creative Commons Attribution 4.0 International license.

**FIG 4:**
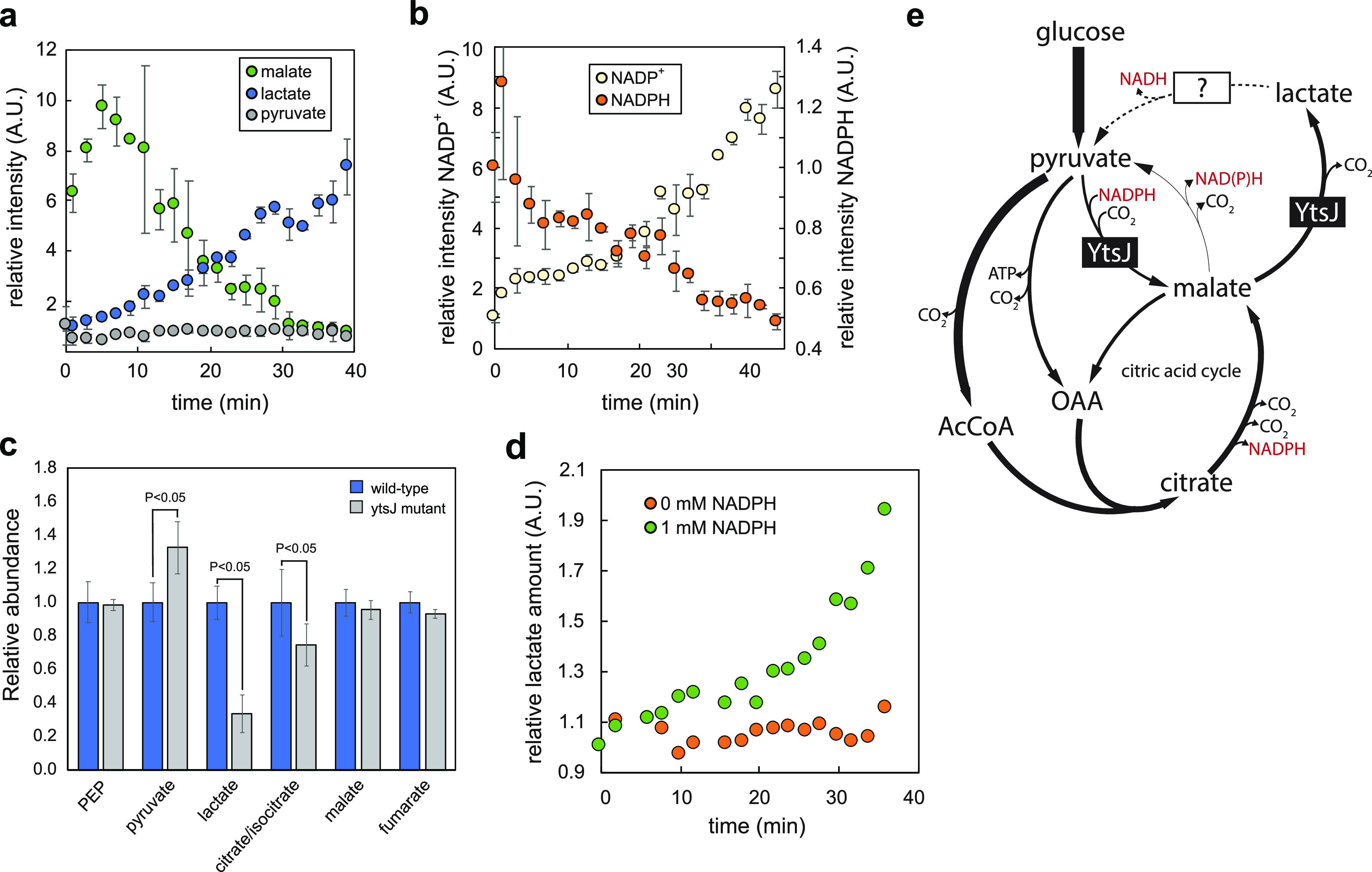
NADPH-induced malolactic activity of malic enzymes. (a and b) Time courses of relative intensities of significantly changing metabolites in the assay with YtsJ, where no malate was added to the mixture. Error bars represent standard deviations calculated using two independent assays. (c) Relative abundance and standard deviations of intracellular metabolites during mid-exponential growth phase of B. subtilis wild type and the *ytsJ* deletion mutant, estimated by targeted LC-MS/MS analysis. Concentration values are normalized to concentrations in the wild type. *P* < 0.05 (paired Student’s *t* test, unequal variance). (d) Time course of relative intensities of lactate in the assay with 0 and 1 mM NADPH, respectively. (e) Proposed role of YtsJ in NADPH homeostasis in B. subtilis.

To confirm that lactate formation occurred directly from malate and not through the subsequent reduction of pyruvate, we repeated the *in vitro* assay with YtsJ in the presence of NAD^+^, NADPH, and pyruvate but without malate. We observed that pyruvate converted first to malate (consuming NADPH), and lactate production followed at a later point (Fig. 4a and b). These results confirm that YtsJ catalyzed malate decarboxylation to lactate and CO_2_ independent of cofactors. This activity is typically found in lactic acid bacteria, where so-called malolactic enzymes catalyze the reaction without release of NAD(P)H (30).

### Malolactic YtsJ activity is also present *in vivo*.

Next, we tried to collect evidence for *in vivo* malolactic activity. Because of identical atom mapping of several involved reactions, we could not devise a selective labeling strategy to quantify malolactic flux. Instead, we resorted to metabolomics to test whether YtsJ is indeed involved in lactate formation. The expectation was that in the Δ*ytsJ* mutant, a major source of lactate is missing; therefore, intracellular levels should drop. We compared steady-state metabolite levels of the wild type and the Δ*ytsJ* deletion mutant during exponential growth on glucose minimal medium. While most detected metabolites were unaffected, lactate abundance was indeed substantially decreased and pyruvate abundance increased in the Δ*ytsJ* mutant (Fig. 4c), which is consistent with the lack of YtsJ-dependent pyruvate carboxylation but also could result from the reduced TCA cycle flux measured upon deletion of *ytsJ*. No extracellular lactate could be detected. We speculated that lactate dehydrogenase (Ldh) routes lactate back to pyruvate, donating electrons to NADH formation (31). This would *de facto* reconstitute a transhydrogenation cycle to convert excess NADPH into NADH. However, fluxes in a Δ*ldh* mutant were similar to those of the wild type (data not shown), indicating that other mechanisms or enzymes act to metabolize lactate. Overall, *in vitro* and *in vivo* evidence demonstrated that YtsJ is bifunctional with both malic and malolactic enzyme activity.

### NADPH also induces malolactic activity for E. coli MaeB.

It was reported before that YtsJ has a characteristic primary protein sequence that differs from that of NAD-dependent isoenzymes (8). Based on sequence homology, most bacterial species contain at least one *ytsJ* homolog. Normally, these are annotated as malic enzymes. We wondered whether homologs also feature NADPH-dependent malolactic activity and chose E. coli’s MaeB (9) as a representative. We performed a mass spectrometry-based transhydrogenation assay, incubating purified MaeB with NAD^+^, malate, and pyruvate. In the presence of 1 mM NADPH, we observed lactate production and, thus, malolactic activity by MaeB (Fig. 4d). Therefore, we speculate that the dual activity is conserved across YtsJ homologs.

## DISCUSSION

We discovered that YtsJ is a bifunctional enzyme. First, it catalyzes the reversible oxidative carboxylation of l-malate to pyruvate, employing NADP^+^ as an electron acceptor (EC 1.1.1.40). This is the well-described activity that is attributed to YtsJ (8) and its homologs across species. We demonstrated that YtsJ (and its homolog, MaeB from E. coli) also catalyzes the nonoxidative decarboxylation of malate to lactate (EC 4.1.1.101). In our *in vitro* tests with physiological concentrations of reactants, both activities were substantial. Therefore, we conclude that it is more appropriate to define YtsJ as bifunctional rather than a promiscuous enzyme with collateral side activity (32, 33).

Coexistence of partly competing activities would be problematic *in vivo*. This raises the question of regulation. We found that NADPH is a strong modulator that triggers the switch between oxidative and nonoxidative decarboxylation. *In vitro*, malolactic activity was clearly detectable with 0.1 mM NADPH. Considering that the physiological NADPH concentration is higher (0.36 ± 0.25 mM) (14, 25), we expect it to be normally present *in vivo*. This claim is confirmed by the drop in lactate observed in the Δ*ytsJ* deletion mutant. However, *in vitro* we also saw that maximum malolactic activity required much higher NADPH levels of 1 mM or more. In summary, it appears that YtsJ has evolved to avoid excessive NADPH production through oxidative malate decarboxylation when NADPH is already abundant. With increasing NADPH, nonoxidative decarboxylation to lactate takes over. It seems plausible that lactate is further oxidized to pyruvate to obtain an overall flux identical to that of malic enzyme. Even though we did not identify the dehydrogenase catalyzing this reaction, we speculate that it is associated with NAD^+^ reduction to not burden NADPH levels, which would constitute a full transhydrogenation cycle.

In the case of B. subtilis growing on glucose, our data prove that the contribution of YtsJ to NADPH balancing goes well beyond avoiding excessive accumulation. The quantitative ^13^C-metabolic flux analysis demonstrated that there is substantial reverse flux of the malic enzyme reaction from pyruvate to malate. Since this reaction proceeds in the reductive direction and oxidizes NADPH (Fig. 4e), it assumes an active role in balancing NADPH that has been produced in excess by other reactions, i.e., isocitrate dehydrogenase. As the measured affinity of YtsJ for NADPH is relatively low (*K_m_*, 0.8 mM), the reductive reaction is likely to occur only with abundant NADPH. Under these conditions, however, the impact on NADPH balancing is major. If lactate-to-pyruvate conversion was coupled to NAD^+^ reduction, a full NADPH-to-NADH transhydrogenation cycle would be complete. Such a cycle would be neutral in terms of carbon and energy and, therefore, proceed as long as thermodynamics are favorable (e.g., NADPH is in excess). YtsJ also has an important role in the opposite scenario of catabolic NADPH underproduction. The extreme case is growth in malate minimal medium, in which the isocitrate dehydrogenase and oxidative pentose phosphate pathway fluxes are very low and YtsJ is essential for fulfilling biosynthetic NADPH demand (25). Not surprisingly, YtsJ is expressed constitutively and oxidative decarboxylation proceeds, driven by thermodynamics and kinetics (34). Overall, NADPH homeostasis does not require transcriptional regulation; thus, the system can adjust instantaneously and on demand. This rapid mechanism is also advantageous to immediately meet a suddenly increased NADPH demand, i.e., upon acute oxidative stress.

Sequence homologs of YtsJ are well represented across bacterial species (8, 35). For E. coli’s homolog MaeB, our *in vitro* tests confirmed that two key characteristics of YtsJ are conserved: it possesses malolactic activity in the presence of NADPH. However, it is unlikely that the identified mechanism of NADPH oxidation is of relevance in E. coli. First, E. coli has been shown to produce little NADPH, which is compensated for by the membrane-bound transhydrogenase PntAB at the cost of proton-motive force (5, 14). Second, occasional overproduction is efficiently resolved by the soluble form of transhydrogenase. Instead, physiological relevance should be tested in organisms that are prone to catabolic overproduction and lack genetic evidence for a transhydrogenase, such as Paracoccus versutus or Zymomonas mobilis (14). The study would require an in-depth analysis of metabolic fluxes and catabolic NADPH production in response to a loss of YtsJ.

## MATERIALS AND METHODS

### Bacterial strains, growth conditions, and media.

The E. coli strains used for overproduction of malic enzymes from B. subtilis (His_6_-MaeA, His_6_-MalS, His_6_-MleA, and His_6_-YtsJ [8]) were grown in Luria-Bertani (LB) medium supplemented with 100 mg liter^−1^ ampicillin and 25 mg liter^−1^ kanamycin. E. coli MaeB enzyme was overexpressed using the respective strain from the ASKA His tag collection library (36) grown in LB medium supplemented with 5 g/liter glucose and 20 μg/ml chloramphenicol.

The B. subtilis mutant strains used in this study are listed in Table S3 in the supplemental material. Frozen glycerol stocks were used to inoculate 5 ml of LB medium. Antibiotics for selection were added at 5 mg liter^−1^ (chloramphenicol), 0.4 mg liter^−1^ (erythromycin), 5 mg liter^−1^ (kanamycin), 100 mg liter^−1^ (ampicillin), or 100 mg liter^−1^ (spectinomycin). After 5 h of incubation at 37°C and 300 rpm on a gyratory shaker, 5 ml of M9 minimal medium was inoculated at 1,000- to 8,000-fold dilutions as precultures. Mid-exponential M9 precultures at optical densities at 600 nm (OD_600_) of 1 to 2 were used to inoculate 35-ml M9 batch cultures in 500-ml shake flasks to an OD_600_ of 0.03. The M9 medium contained, per liter of deionized water, 8.5 g of Na_2_HPO_4_ 2H_2_O, 3.0 g KH_2_PO_4_, 1 g NH_4_Cl, 0.5 g NaCl and was adjusted to pH 7 before autoclaving. The following components were filter sterilized separately and then added (per liter of final medium): 1 ml 1 M MgSO_4_, 1 ml 0.1 M CaCl_2_, 1 ml 0.05 M FeCl_3_, and 10 ml of a trace element solution containing (per liter) 170 mg ZnCl_2_, 100 mg MnCl_2_ 4H_2_O, 60 mg CoCl_2_ 6H_2_O, 60 mg Na_2_MoO_4_ 2H_2_O, and 43 mg CuCl_2_ 2H_2_O. Autoclaved carbon source solutions were added to a final concentration of 5 g liter^−1^.

10.1128/mBio.03438-20.6 TABLE S3 B. subtilis strains used in this study. Download Table S3, PDF file, 0.2 MB. Copyright © 2021 Hörl et al. 2021 Hörl et al. https://creativecommons.org/licenses/by/4.0/ This content is distributed under the terms of the Creative Commons Attribution 4.0 International license.

### Physiological parameters.

Cell growth was determined spectrophotometrically at 600 nm. Glucose, acetate, fumarate, pyruvate, lactate, and succinate concentrations in the supernatant were estimated by the signals of a refractive index and diode array detector on a high-performance liquid chromatograph (HPLC; Agilent 1100), using an Aminex HPX-87H column at a temperature of 60°C with 5 mM H_2_SO_4_ as the eluent. Supernatant samples were prepared by centrifugation of 1 ml culture broth for 5 min at 4°C and 14,000 × *g*. Specific growth rates were calculated by linear regression of logarithmic OD_600_ over time. Specific uptake and secretion rates were estimated by linear regression of the substrate or product concentration against biomass concentration.

### Stationary ^13^C metabolic flux analysis.

Fluxes in the central metabolism of B. subtilis were estimated according to Zamboni et al. (22). Cultures were inoculated to an OD_600_ of 0.03 in M9 medium containing 100% [1-^13^C]glucose and a mixture of 50% (wt/wt) uniformly labeled and 50% naturally labeled glucose. During the mid-exponential growth phase, 1 ml of cell broth was harvested by centrifugation (2 min, 23,000 × *g*, 4°C), washed with 0.9% NaCl, and stored at −20°C until further analysis. The pellets were hydrolyzed with 6 M HCl at 105°C for 18 h and dried at 95°C under a constant air stream. Hydrolysates were dissolved in 20 μl of dimethylformamide (Sigma-Aldrich) and transferred to GC-MS vials. After the addition of 20 μl *N*-tertbutyldimethylsilyl-*N*-methyltrifluoroacetamide with 1% (wt/wt) tertbutyldimethyl-chlorosilane (Sigma-Aldrich), the mixture was incubated at 85°C for 1 h. Subsequently, mass isotopomer distributions of protein-bound amino acids were determined on a 6890N GC system (Agilent Technologies) combined with a 5875 Inert XL MS system (Agilent Technologies).

After correction for naturally occurring stable isotopes, amino acid mass isotopomer distributions were used to calculate ratios of converging metabolic fluxes. These ratios, together with extracellular fluxes and a stoichiometric model of B. subtilis central metabolism (23), were then used as constraints to calculate absolute intracellular fluxes. All calculations were performed using FiatFlux (37).

### Intracellular metabolite measurements.

B. subtilis strains were grown in a shake flask culture to mid-exponential phase (OD_600_ between 0.5 and 1). An amount proportional to 1 ml OD of the culture broth was transferred onto a 0.45-μm-pore-size Durapore filter (Millipore) and vacuum filtered, and the filter was immediately transferred into 4 ml of −20°C acetonitrile-methanol-water (2:2:1) to quench metabolism and kept at −20°C for 1 h for extraction. For pyruvate measurements, 2 ml OD was similarly sampled by filtration and added to the acetonitrile-methanol-water mixture containing 25 μM phenylhydrazine for derivatization of α-keto acids (38). Experiments were performed in triplicates with cells from different shake flasks.

The supernatants were dried at 12 Pascal in a SpeedVac composed of an Alpha 2–4 LD plus cooling trap, an RVC 2–33 rotational vacuum concentrator, and an RC-5 vacuum chemical hybrid pump (Christ, Osterode am Harz, Germany). Dried extracts were resuspended in 100 μl deionized water, 10 μl of which was injected into a Waters Acquity UPLC with a Waters T3 column (150 by 2.1 mm by 1.8 μm; Waters Corporation, Milford, MA, USA) coupled to a Thermo TSQ Quantum Ultra triple-quadrupole instrument (Thermo Fisher Scientific, Waltham, MA, USA) with electrospray ionization. Compound separation and acquisition were achieved as described previously (29, 38).

### Malic enzyme expression and purification.

His_6_-tagged proteins from B. subtilis were overexpressed using the QIAexpress kit (Qiagen), inducing with 1 mM isopropyl-β-d-thiogalactopyranoside (IPTG) for 5 to 7 h. MaeB from E. coli was directly overexpressed in the growth medium by adding 0.2 mM IPTG. Cells were harvested by centrifugation at 4°C, washed with 0.9% NaCl, resuspended in lysis buffer (100 mM Tris-HCl, pH 7.5, 5 mM MgCl_2_, 1 mM dithiothreitol [DTT], and 4 mM phenylmethylsulfonyl fluoride), and disrupted by three passages through a French press cell at 4°C. Cell-free lysates were obtained by centrifugation for 10 min at 23,000 × *g* and 4°C. His_6_-tagged proteins were purified using Ni^2+^-charged nitrilotriacetic acid (Ni-NTA) affinity columns (GE Healthcare) according to the manufacturer’s instructions. Proteins were eluted from the column with elution buffer (500 mM imidazole), which was subsequently replaced by storage buffer (50 mM Tris, pH 8, 150 mM NaCl, 1 mM DTT, and 0.5 mM EDTA) (8) using ultrafiltration columns with a 10-kDa size cutoff (Millipore). The correct size was verified by SDS-PAGE. Proteins were stored at 4°C for, at most, 1 day before performing activity assays.

### Spectrophotometric activity assays.

Reverse malic enzyme activities were tested at 37°C by spectrophotometrically monitoring NAD(P)H oxidation [pyruvate plus NAD(P)H into malate plus NAD(P)^+^] at 340 nm. The reaction mixture consisted of 100 mM Tris-HCl, pH 7.8, 5 mM MgCl_2_, 50 mM KCl, 50 mM NaHCO_3_, 20 mM pyruvate, and 0.2 mM NAD(P)H (14). For determination of the *K_m_* for pyruvate, both cofactors NADH and NADPH were added to the reaction mixture at 0.2 mM. The *K_m_* for cofactors was estimated at a saturating pyruvate concentration of 20 mM. For both cofactors, a molar extinction coefficient of 6.22 × 10^6^ cm^2^ mol^−1^ was used for calculations. To determine *K_m_* and *k*_cat_, the initial reaction rates were fitted to a Michaelis-Menten relationship by least-squares analysis. Enzyme concentrations were estimated by a Bradford protein assay (39).

### Mass spectrometry-based transhydrogenation assays.

Purified YtsJ and MaeB were incubated at room temperature in 200 μl of 10 mM potassium phosphate buffer, pH 7.4, 2.5 mM MgCl_2_, 50 mM KCl, and 1 mM NaHCO_3_ with a mixture of NAD^+^, malate, and pyruvate at physiological *in vivo* concentrations (Table S4) and various amounts of NADPH. The enzyme reaction samples were assayed by direct online flow injection into a TOF MS (6520 Series QTOF; Agilent Technologies) operated in the negative ionization mode. High-precision mass spectra were recorded from *m/z* 50 to 1,000 and analyzed as described previously (28). Except for NADPH, the relative intensities for all metabolites were calculated by normalizing the intensities of each time course to the initial intensity at 0 min for each NADPH level. For NADPH, the relative intensity was calculated by normalizing all values to the initial intensity at 0 min of the assay when no NADPH was added to the mixture.

10.1128/mBio.03438-20.7 TABLE S4 Physiological concentrations of metabolites involved in the malic enzyme reaction in B. subtilis. Download Table S4, PDF file, 0.1 MB. Copyright © 2021 Hörl et al. 2021 Hörl et al. https://creativecommons.org/licenses/by/4.0/ This content is distributed under the terms of the Creative Commons Attribution 4.0 International license.

### Thermodynamic analysis.

The Gibbs free energies (Δ_r_*G*) of NAD^+^-dependent malate decarboxylation (EC 1.1.1.38) and NADPH-dependent pyruvate carboxylation (EC 1.1.1.40) were calculated using the EQuilibrator software (http://equilibrator.weizmann.ac.il) (26). To account for the effect of metabolite concentrations on the Gibbs free energies, published physiological metabolite concentrations during exponential growth of B. subtilis on glucose were entered for each reaction (14, 25) (Table S2). For the CO_2_ level, a concentration of 10 μM was assumed, which represents the amount of dissolved CO_2_ under atmospheric conditions (40). The pH and ionic strength were left at the standard settings of 7 and 0.1 M, respectively. The standard deviations due to concentration measurement errors were 6 kJ/mol for NAD^+^-dependent malate decarboxylation and 8 kJ/mol for NADPH-dependent pyruvate carboxylation. The standard error due to assumptions in the EQuilibrator software was 6.2 kJ/mol.

### Data availability.

All data have been deposited in BioStudies (accession no. S-BSST566) and MetaboLights (accession no. MTBLS2304) databases.
