# Supplementary material for: Bifunctional Malic/Malolactic Enzyme Provides a Novel Mechanism for NADPH-Balancing in Bacillus subtilis
Source: mBio. 2021 Apr 6;12(2):e03438-20. doi: 10.1128/mBio.03438-20 (PMC8092299; doi:10.1128/mBio.03438-20)
Supplement: FIG S1 [file mBio.03438-20-sf001.pdf]

Supplementary Figure 1. Metabolic flux distributions in *B. subtilis* malic enzyme deletion mutants, estimated by ^{13}C flux ratio analysis and metabolite balancing. Flux values are normalized to the glucose uptake rate (q_{glc}, mmol g_{CDW}^{-1} h^{-1}) of each strain. Arrow dimensions scale with flux magnitude.

[figure on next page]

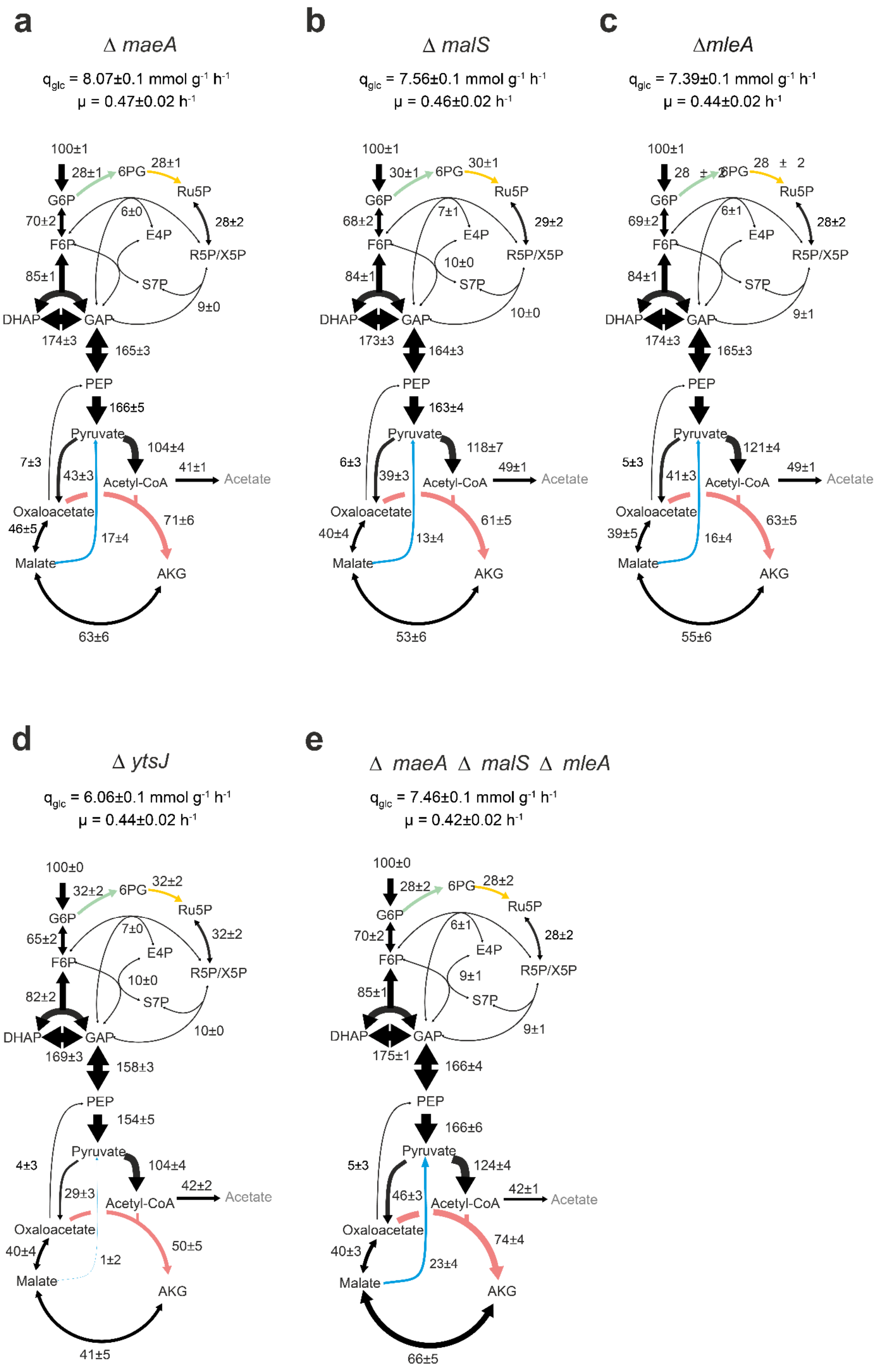

a
Δ maeA
qglc = 8.07±0.1 mmol g-1 h-1
μ = 0.47±0.02 h-1
100±1
28±1
6PG
28±1
Ru5P
G6P
6±0
70±2
E4P
28±2
F6P
R5P/X5P
85±1
S7P
9±0
DHAP
GAP
174±3
165±3
PEP
166±5
Pyruvate
104±4
7±3
43±3
Acetyl-CoA
41±1
Acetate
Oxaloacetate
71±6
46±5
17±4
Malate
AKG
63±6
b
Δ malS
qglc = 7.56±0.1 mmol g-1 h-1
μ = 0.46±0.02 h-1
100±1
30±1
6PG
30±1
Ru5P
G6P
7±1
68±2
E4P
29±2
F6P
10±0
R5P/X5P
84±1
S7P
10±0
DHAP
GAP
173±3
164±3
PEP
163±4
Pyruvate
118±7
6±3
39±3
Acetyl-CoA
49±1
Acetate
Oxaloacetate
61±5
40±4
13±4
Malate
AKG
53±6
c
ΔmleA
qglc = 7.39±0.1 mmol g-1 h-1
μ = 0.44±0.02 h-1
100±1
28 ± 2
6PG
28 ± 2
Ru5P
G6P
6±1
69±2
E4P
28±2
F6P
R5P/X5P
84±1
S7P
9±1
DHAP
GAP
174±3
165±3
PEP
Pyruvate
121±4
5±3
41±3
Acetyl-CoA
49±1
Acetate
Oxaloacetate
63±5
39±5
16±4
Malate
AKG
55±6
d
Δ ytsJ
qglc = 6.06±0.1 mmol g-1 h-1
μ = 0.44±0.02 h-1
100±0
32±2
6PG
32±2
Ru5P
G6P
7±0
65±2
E4P
32±2
F6P
10±0
R5P/X5P
82±2
S7P
10±0
DHAP
GAP
169±3
158±3
PEP
154±5
Pyruvate
104±4
4±3
29±3
Acetyl-CoA
42±2
Acetate
Oxaloacetate
50±5
40±4
1±2
Malate
AKG
41±5
e
Δ maeA Δ malS Δ mleA
qglc = 7.46±0.1 mmol g-1 h-1
μ = 0.42±0.02 h-1
100±0
28±2
6PG
28±2
Ru5P
G6P
6±1
70±2
E4P
28±2
F6P
9±1
R5P/X5P
85±1
S7P
9±1
DHAP
GAP
175±1
166±4
PEP
166±6
Pyruvate
124±4
5±3
46±3
Acetyl-CoA
42±1
Acetate
Oxaloacetate
74±4
40±3
23±4
Malate
AKG
66±5
