# Supplementary material for: Bifunctional Malic/Malolactic Enzyme Provides a Novel Mechanism for NADPH-Balancing in Bacillus subtilis
Source: mBio. 2021 Apr 6;12(2):e03438-20. doi: 10.1128/mBio.03438-20 (PMC8092299; doi:10.1128/mBio.03438-20)
Supplement: TABLE S1 [file mBio.03438-20-st001.pdf]

Supplementary Table 1. Physiological parameters of the six *B. subtilis* strains used in this study. The mean values and standard deviations were determined from at least duplicate experiments.

Strain	Genotype	Growth rate (h-1)	Glucose uptake rate (mmol g-1 h-1)	Acetate secretion rate (mmol g-1 h-1)	Yield (gCDW/g glucose)
168CA	wild-type	0.51 ± 0.02	8.91 ± 1.4	3.22 ± 0.70	0.32
GM1608	ΔytsJ	0.44 ± 0.02	6.06 ± 0.79	2.75 ± 0.35	0.40
GTD102	ΔmaeA	0.47 ± 0.03	8.07 ± 0.99	3.30 ± 0.31	0.32
GTD110	ΔmalS	0.46 ± 0.02	7.56 ± 0.71	3.69 ± 0.25	0.34
GM1632	ΔmleA	0.44 ± 0.03	7.39 ± 1.20	3.59 ± 0.40	0.33
GM1655	ΔmaeAΔmalSΔmleA	0.42 ± 0.01	7.46 ± 1.42	3.23 ± 0.64	0.31
