# Supplementary material for: Bifunctional Malic/Malolactic Enzyme Provides a Novel Mechanism for NADPH-Balancing in Bacillus subtilis
Source: mBio. 2021 Apr 6;12(2):e03438-20. doi: 10.1128/mBio.03438-20 (PMC8092299; doi:10.1128/mBio.03438-20)
Supplement: TABLE S2 [file mBio.03438-20-st002.pdf]

Supplementary Table 2. Enzymatic parameters of the four *B. subtilis* malic enzyme. K_m values are provided in mM, k_{cat} values in s^{-1}. The mean values and standard deviation were determined with at least 2 replicates.

Protein	Pyruvate[a]		NADH[a]		NADPH[a]		Malate[b]		NAD^{+b}		$NADP^{+b}$	
	K_m	k_{cat}	K_m	k_{cat}	K_m	k_{cat}	K_m	k_{cat}	K_m	k_{cat}	K_m	k_{cat}
MaeA	nd[c]	nd	nd	nd	nd	nd	1.55	54	2.80	3	0.055	4
MalS	nd	nd	nd	nd	nd	nd	1.56	47	1.00	9	nd	nd
MleA	nd	nd	nd	nd	nd	nd	3.52	56	5.50	495	7.3	66
YtsJ	5.3±1.3	4.3±0.4	nd	nd	0.8±0.5	7.8±2.7	3.95	75	5.30	159	6.7	54

[a] this study

[b] Lerondel et al. (2006). The maximal deviation from the mean was less than 10%[8].

[c] not detected
