# Supplementary material for: Bifunctional Malic/Malolactic Enzyme Provides a Novel Mechanism for NADPH-Balancing in Bacillus subtilis
Source: mBio. 2021 Apr 6;12(2):e03438-20. doi: 10.1128/mBio.03438-20 (PMC8092299; doi:10.1128/mBio.03438-20)

**Supplementary Figure 2.** K-means clustering of the log-transformed annotated ion responses of both assays with 1mM NADPH, normalized to the initial time point. Three clusters were specified and ions were assigned to the clusters based on squared Euclidean distance. The black line represents the centroid of each cluster. Cluster 1 of strongly increasing ions contained lactate and $NADP^+$, cluster 3 of strongly decreasing ions contained malate as deprotonated (m/z = 133) ion and an ion with neutral loss of water (m/z = 115).

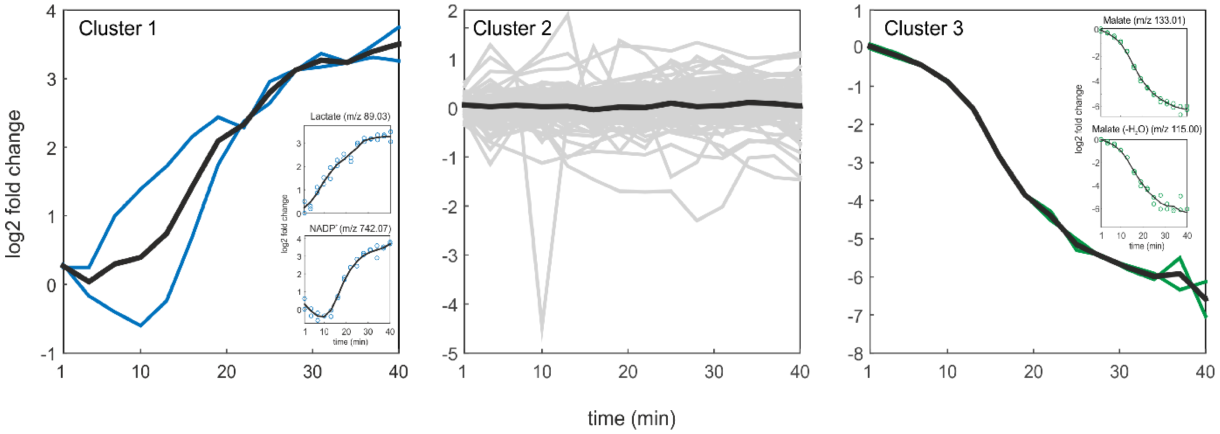

Supplement: FIG S2 [file mBio.03438-20-sf002.pdf]
