# Supplementary material for: Bifunctional Malic/Malolactic Enzyme Provides a Novel Mechanism for NADPH-Balancing in Bacillus subtilis
Source: mBio. 2021 Apr 6;12(2):e03438-20. doi: 10.1128/mBio.03438-20 (PMC8092299; doi:10.1128/mBio.03438-20)

Supplementary Figure 3. Confirmation of ion m/z = 89 in enzyme assay mixture as lactate by MRM analysis (89>43 transition) using targeted LC-MS/MS.

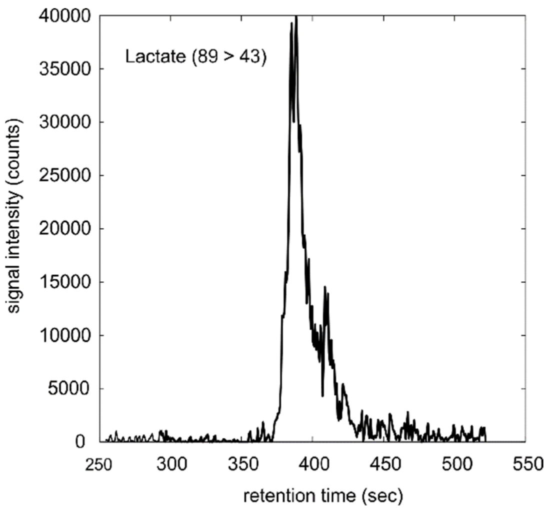

Supplement: FIG S3 [file mBio.03438-20-sf003.pdf]
