# Supplementary material for: Bifunctional Malic/Malolactic Enzyme Provides a Novel Mechanism for NADPH-Balancing in Bacillus subtilis
Source: mBio. 2021 Apr 6;12(2):e03438-20. doi: 10.1128/mBio.03438-20 (PMC8092299; doi:10.1128/mBio.03438-20)
Supplement: TABLE S3 [file mBio.03438-20-st003.pdf]

Supplementary Table 3. ***B. subtilis*** **strains used in this study**

Strain	Genotype	Source or reference
168CA	wild-type, *trpC2*	Laboratory stock
GM1608	*ytsJ'*::[pMUTIN2 Δ(*lacZ-ery*)::*kan*]	(Lerondel et al., 2006)[8]
GTD102	*maeA*'::*kan*	(Doan et al., 2003)[42]
GTD110	Δ*malS*'::*spc*	(Doan et al., 2003)[42]
GM1632	*mleA'*::*cat*	(Lerondel et al., 2006)[8]
GM1655	*maeA::pEC23 (kan) malS::spec mleA::pMutin3 (ery)*	(Lerondel et al., 2006)[8]
