# Supplementary material for: Bifunctional Malic/Malolactic Enzyme Provides a Novel Mechanism for NADPH-Balancing in Bacillus subtilis
Source: mBio. 2021 Apr 6;12(2):e03438-20. doi: 10.1128/mBio.03438-20 (PMC8092299; doi:10.1128/mBio.03438-20)
Supplement: TABLE S4 [file mBio.03438-20-st004.pdf]

Supplementary Table 4. Physiological concentrations of metabolites involved in the malic enzyme reaction in *B. subtilis*.

metabolite	Concentration (mM)	Standard deviation
Malate	0.57[a]	0.14
Pyruvate	8.90[a]	5.75
NAD^+	1.09[b]	0.52
NADH	0.43[b]	0.21
$NADP^+$	0.19[b]	0.14
NADPH	0.36[b]	0.25

[a] Estimates from Kleijn et. al. (2010)[25]. [b] Concentrations calculated from the determined cofactor ratios in Fuhrer et al. (2009)[14] and absolute pool sizes of combined NAD^+/NADH and $NADP^+$/NADPH[25].
